# Quantifying variability of intrafractional target motion in stereotactic body radiotherapy for lung cancers

**DOI:** 10.1120/jacmp.v14i5.4319

**Published:** 2013-09-06

**Authors:** Mark K.H. Chan, Dora L.W. Kwong, Eric Tam, Anthony Tong, Sherry C.Y. Ng

**Affiliations:** ^1^ Department of Clinical Oncology The University of Hong Kong Hong Kong China; ^2^ Department of Clinical Oncology Tuen Mun Hospital Hong Kong China; ^3^ Department of Clinical Oncology Queen Mary Hospital Hong Kong China; ^4^ Theresa Po CyberKnife Center Hong Kong China

**Keywords:** intrafractional variation of tumor motion, stereotactic body radiotherapy

## Abstract

In lung stereotactic body radiotherapy (SBRT), variability of intrafractional target motion can negate the potential benefits of four‐dimensional (4D) treatment planning that aims to account for the dosimetric impacts of organ motion. This study used tumor motion data obtained from CyberKnife SBRT treatments to quantify the reproducibility of probability motion function (pmf) of 37 lung tumors. The reproducibility of pmf was analyzed with and without subtracting the intrafractional baseline drift from the original motion data. Statistics of intrafractional tumor motion including baseline drift, target motion amplitude and period, were also calculated. The target motion amplitude significantly correlates with variations (1 SD) of motion amplitude and baseline drift. Significant correlation between treatment time and variations (1 SD) of motion amplitude was observed in anterior‐posterior (AP) motion, but not in craniocaudal (CC) and left‐right (LR) motion. The magnitude of AP and LR baseline drifts significantly depend on the treatment time, while the CC baseline drift does not. The reproducibility of pmf as a function of time can be well described by a two‐exponential function with a fast and slow component. The reproducibility of pmf is over 60% for the CC motion and over 50% for the AP and LR motions when baseline variations were subtracted from the original motion data. It decreases to just over 30% for the CC motion and about 20% for the AP and LR motion, otherwise. 4D planning has obvious limitations due to variability of intrafractional target motion. To account for potential risks of overdosing critical organs, it is important to simulate the dosimetric impacts of intra‐ and interfractional baseline drift using population statistics obtained from SBRT treatments.

PACS number: 87.55.‐x

## I. INTRODUCTION

Four‐dimensional (4D) treatment planning aims to account for the effects of internal organ motion in lung radiotherapy. It has been demonstrated that the dosimetric effect of target motion can be modeled by convolving the probability motion function (pmf) with the static dose distribution or directly incorporating the pmf into the optimization problem.^(^ ^1^ ^,^ ^2^ ^)^ However, these convolution‐based methods do not deal with organ deformation during respiration. The other common approach is 4D dose calculation. In this approach, dose distributions are calculated and/or optimized on multiple breathing geometries obtained from a 4D computed tomography (4D CT) dataset, and summed together using deformable image registration. The resulting 4D dose distribution explicitly accounts for the cumulative effect of temporal changes of anatomic structures.^(^ ^3^ ^,^ ^4^ ^)^ Such 4D planning technique is particularly useful to incorporate the additional movement of beam aperture in dynamic multileaf (MLC)‐based or robotic‐based target tracking radiotherapy.^(^ ^5^ ^,^ ^6^ ^)^

The robustness of any 4D planning regime depends on the reliability of the motion model input. In fact, study using repeated 4D CT has demonstrated that tumor mobility increased between weeks during the treatment course.^(7)^ Experiences from image‐guided (IG) radiotherapy also indicated that target exhibited changes of characteristics of the periodic (cyclic) respiration‐induced motion and nonperiodic (ultracyclic) baseline motion, in spite of rather stable tumor trajectory between treatment fractions.^(8)^ The random and systematic uncertainties of interfractional target position effectively vary the pmf and, consequently, the dose distribution.

For stereotactic body radiotherapy (SBRT) of lung cancers, intrafractional variation of tumor motion may also be important due to the complex setup procedures and the long beam‐on time leading to an increase of overall treatment duration. Purdie et al.^(9)^ acquired cone‐beam computed tomography (CBCT) before and after treatment delivery to assess the intrafractional errors of target positions, which included contributions from overall patient movement and target baseline drift. They found a correlation between the target position error and the treatment time. Similarly, Sonke et al.^(10)^ used respiratory‐correlated CBCT (rCBCT) to quantify the intrafractional target position errors relative to bony anatomy (i.e., intrafractional baseline drift). They found systematic errors (1 SD) of 1.0, 1.4, and 0.6 mm, and random errors of 1.1, 1.5, 0.7 mm in the CC, AP, and LR directions, respectively. Due to the constant treatment time, they did not observe a correlation between the drift of tumor baseline and the treatment time. On the other hand, Suh et al.^(11)^ used continuous tumor motion data extracted from CyberKnife SBRT treatments to obtain statistics of intrafractional tumor motion. They estimated the overall mean cycle‐to‐cycle variations of tumor motion amplitude and period to be 0.2 cm and 0.8 s, respectively. However, none of these studies (and others) quantified how the variations of tumor motion amplitude and period and baseline affect the reproducibility of pmf.^(12)^ Cai et al.^(13)^ have employed dynamic MRI to study the reproducibility of interfractional lung pmf. However, their motion data was limited to 300 s, which were much shorter than the typical length of actual SBRT treatments (one‐half to one hour, and even up to two hours in CyberKnife real‐time target tracking SBRT). It is the aim of this study to assess the intrafractional variations of tumor motion and the reproducibility of the pmf on a times scale that corresponds to actual SBRT treatments. It is expected that results obtained in this study shall shed insight into the appropriateness of 4D planning of SBRT for lung cancers.

## II. MATERIALS AND METHODS

### A. Acquisition and analysis of tumor motion data

Between 2008 and 2011, 37 lesions (15 lower‐lobe tumors, 14 middle‐lobe tumors, and eight upper‐lobe tumors) of a total of 28 patients were treated with SBRT using the CyberKnife robotic radiosurgery system (Accuray Inc., Sunnyvale, CA). There were a total of 128 treatment fractions. The target motion data of these patients were obtained from the log files of the Synchrony Respiratory Tracking System (RTS) (Accuray Inc. Sunnyvale, CA). Note that the Synchrony RTS does not track the tumor itself, but records the center‐of‐mass (COM) of the implanted fiducials as computed by the pair of stereoscopic images. For an elaboration of technical details about the tracking mechanism, see Seppenwoolde et al.^(6)^ and Hoogeman et al.^(14)^ The motion log file contained motion data in three principal directions in the format of t:x,y,z, where *t* is the time in seconds, *x, y*, and *z* are the target positions in millimeter (mm) in the craniocaudal (CC), left‐right (LR), and anterior‐posterior (AP) directions. Note that the target motion was based on the estimate of the correlation model that was built using the external breathing signal and the internal fiducials COM's position. Therefore, it did not strictly represent the true target positions. The mean correlation model errors have been estimated to be better than 0.3 mm.^(14)^

**Figure 1:**
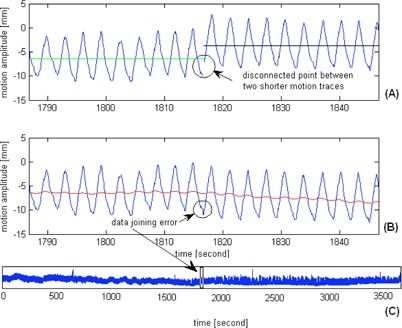
Two motion traces (a) of the same treatment fraction are showed. They were joined by subtracting the difference of the last 30 s average of the motion data before model rebuilt (green solid line) and the first 30 s average of the motion data after model rebuilt (black solid line) from the motion data of the rebuilt model. The datajoining artifact (b) was indicated; the red solid line represents the ultracyclic target baseline motion. The complete time series (c) of target motion after the data joining procedure was demonstrated. With the large number of motion cycles, the artifact of motion data joining is expected to contribute to negligible errors of the overall estimates of target motion amplitude and period.

Prior to the motion analysis, motion traces in each direction were plotted and visually inspected to remove the portions with significant artifacts. To reduce uncertainties of the results, the criteria of exclusion and inclusion of data points recommended by Suh et al.^(11)^ were followed. In our analysis, one fraction could generate more than one motion trace of shorter periods because the correlation model was forced to rebuild whenever the actual and estimated position of the center‐of‐mass of implanted fiducials deviated by 3 mm in a row. Furthermore, since couch movements to reposition the patient were not logged by the target localization system (TLS), there were no direct means to join datasets before and after the couch corrective movements, unless manual efforts were taken to note these movements. In the following analysis, these short motion traces were combined by subtracting the difference of the last 30‐second average of the motion data before model rebuilt and the first 30‐second average of the motion data after model rebuilt from the motion data of the rebuilt model. This data joining procedure was illustrated in Fig. 1. To avoid serious error in the analysis of motion statistics, motion data were combined only if the mean amplitude of the rebuilt motion model was within the mean amplitude ± 2°SD of the beginning motion model. Such criteria was rather arbitrary, but was adopted to exclude obvious error resulting from the motion correlation model while not to overly exclude motion data that might represent the actual change of motion characteristics. Because the motion traces before and after model rebuild may exhibit different amplitudes, errors could occur at the connecting points between the fragmented motion data. However, the resulting errors in the determination of cycle‐to‐cycle period and amplitude were expected to be averaged out given the large number of breathing cycles. Furthermore, the data joining procedure assumed negligible tumor baseline drift before and after intervening couch shifts. This assumption could introduce error of 1.9 mm (1 SD) in the baseline estimation, as showed in a recent study of diaphragm motion during CBCT acquisition.^(15)^ In this work, 20 motion traces were reconstructed by combining motion traces belonging to two correlation models, and three motion traces were generated with traces from three correlation models of the same treatment fraction.

For each motion trace, the peak‐to‐peak amplitude was determined by an in‐house written code in MATLAB (The MathWorks, Inc. Natick, MA). The code included a low‐pass (Gaussian) filter to reduce high frequency noise embedded in the original data, and used the MATLAB built‐in algorithm to locate local maxima. Only motion traces of length > 1000 s were included in this analysis.

### B. Intrafractional variability of tumor motion baseline

Once the portions of the artifacts were removed, the intrafractional variability of the_ultracyclic baseline motion was estimated in a two‐step process: 1) the reference baseline $\overset{‐}{M}$ was established with the first 100 s (starting from the 10th to 110th second) of the motion trace, and 2) a running mean (RM) for the motion traces with a 20 s moving window was created as an approximation of the baseline.

The reason of using 100 s as an estimate of the reference baseline is that it roughly corresponds to the duration needed to build a reliable correlation model with eight evenly distributed points along the breathing cycle (two at either extremes and the other six at the mid‐exhale and mid‐inhale phases), and that it roughly corresponds to the acquisition time of planning 4D CT and verification CBCT. The second step involved extracting the ultracyclic target motion from the motion trace to approximate the time series of mean target positions. For this study, a 20 s moving window was employed to create a RM of the target position inside the window. The evaluation of the baseline drift was started at 110 s (i.e., the next data point to *M)* and stopped at 10 s from the end of the trace.

### C. Intrafractional reproducibility of probability density motion function (pmf)

After the motion data was processed as described above, the pmf was calculated by binning the target displacement, with a constant bin size set to 0.05 mm. The reproducibility of intrafractional pmf was analyzed separately for the CC, AP, and LR target motions. The reference pmf was generated from the same motion data as the reference baseline. It was then compared to the pmfs that were generated every 100 s following the reference pmf to determine the reproducibility R as a function of time t:

(1) $$R(t)=\frac{pmf(t)\cap pmf_{ref}}{pmf(t)\cup pmf_{ref}}$$

The reproducibility of pmf as a function of time R(t) was stopped until the remaining data were < 100 s. In addition, the influence of baseline drift on the reproducibility of intrafractional pmf was also evaluated by subtracting the ultracyclic baseline motion from the original motion data. This resulted in the reproducibility of pmf due to the cyclic tumor motion, Rc(t)

(2) $$R_{c}(t)=\frac{pmf_{c}(t)\cap pmf_{ref}}{pmf_{c}(t)\cup pmf_{ref}}$$

where the subscript *c* indicates the cyclic component of the tumor motion.

### D. Statistical analysis

Statistical correlations between variables were analyzed by linear regression, exponential regression, and ANOVA with two and more factors using the Statistical Toolbox of MATLAB. P‐value for Pearson's correlation (r) resulting from the linear regression was computed using a Student's *t*‐distribution. The Student's *t*‐test was also used to compare groups and variables. A p‐value < 0.05 is considered statistically significant.

## III. RESULTS

A total of 154 motion traces were extracted. The longest and shortest durations were 6000 s and 1200 s (median = 3400 s), respectively. The principal motion coincides with the CC direction. There is an observable trend of increasing CC, AP, and LR motion amplitude in lower lobe tumors than upper and middle lobe tumors (p < 0.05). The overall mean of individual treatment fraction means of target motion amplitude is 6.0 ± 4.6 mm (range: 0.2–18.7 mm), 1.7 ± 1.2 mm (0.2–5.8 mm), and 0.9 ± 0.8 mm (0.0–3.7mm) in the CC, AP, and LR directions, respectively. The overall target motion period is 3.6 ± 0.6 s, with individual treatment fraction means ranging from (2.0–5.6 s). Lengths of the exhale and the inhale period of the patient population range from 0.9–2.2 s (mean: 1.5 s) and 1.1–3.4 s (mean: 2.0 s), respectively.

**Figure 2:**
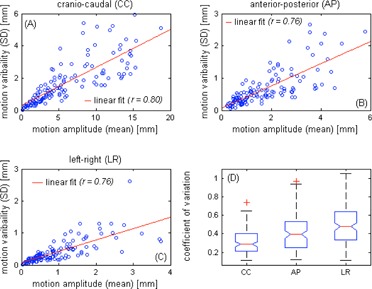
Linear regressions of the motion amplitude and the motion variability (1 SD) in the craniocaudal (a), anterior‐posterior (b), and left‐right (c) directions. Each circle represents each of the 128 motion traces. The linear fits and Pearson's correlation coefficients of the data are also given. Box‐and‐whisker plot (d) of the coefficient of variation (CV) of each motion component is shown. Outliers with a value that is more than 1.5 times the interquartile range away from the top or bottom of the box are marked with ared + sign.

**Figure 3:**
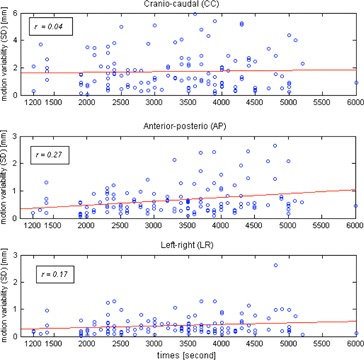
Relationship between variability of motion amplitude (1 SD) and time for each motion axis. Each circle represents each of the 128 motion traces. The red lines are the best fits in the least‐square sense. The Pearson's correlation coefficients *r* are shown.

**Figure 4:**
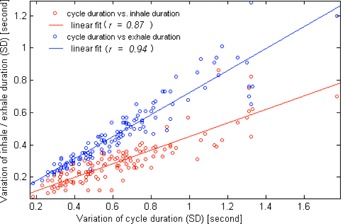
Variation (1 SD) of cycle period was plotted against the variations (1 SD) of the length of exhale (blue dot) and inhale (red dot) states. Each circle represents each of the 128 motion traces. The linear fits and Pearson's correlation coefficients are also given.

In Fig. 2, the linear regressions show increasing variability of intrafractional target motion amplitude (1 SD) with the motion amplitude in all directions ($\text{CC}:\;r\;=\;0.80,p\;<\;10^{-30}$; $\text{AP}:\;r\;=\;0.76,p\;<\;10^{24}$; and $\text{LR}:\;r\;=\;0.76,p\;<\;10^{24}$). From Fig. 3, it was observed that the correlations of the motion amplitude variability and treatment time are moderate for the AP motion and weak for the CC and LR motions. ANOVA indicates that for the CC and LR directions, the variability of motion amplitude significantly depends on the tumor location (p < 0.05) but not on the treatment time (p = 0.98 for CC, and p = 0.073 for LR), whereas for the AP direction the variability depends on both tumor site (p = 0.004) and treatment time (p = 0.002). Variations (1 SD) of the exhale and inhale lengths versus the breathing cycle periods are plotted in Fig. 4.

A slightly higher correlation is observed between the breathing cycle period and the exhale length (r = 0.94) than between thee breathing cycle period and the mhale length (r = 0.87). Statistically, difference of motion period between lower lobe tumors and upper and middle tumors is insignificant (3.5 s vs. 3.7 s; p = 0.08). However, the F‐test shows greater variance of the length of exhehe state than inhale stale $\left(p\;<\;10^{-6}\right)$ of individual treatment fractions.

**Figure 5:**
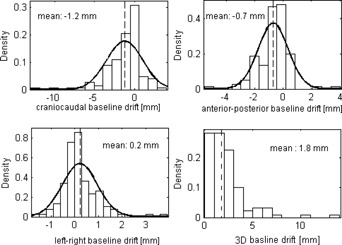
Distribution of the intrafractional baseline drift in each direction. The 3D baseline drift is also given. The solid lines are normal distribution fits and the thin dotted lines indicate means of the baseline drifts.

**Figure 6:**
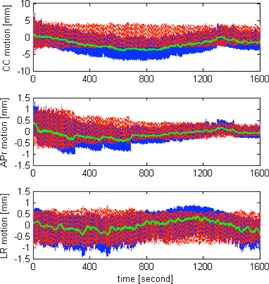
Illustration of the variation of tumor motion in the each direction for one selected patient. The blue solid lines are the original motion data, green lines show the baselines, and the red dotted lines are the original motion data subtracting the baseline (i.e., cyclic motion). The CC baseline drift reaches a minimum of ‐4.0 mm halfway through the treatment and returns to ‐0.2 mm at the end of the treatment.

Figure 5 shows distribution of the baseline drift in each direction and the distribution of the 3D baseline drift. The population statistics in terms of group mean, systematic, and random errors are summarized in Table 1. Drifts are predominately towards the patients' superior (or exhale), posterior, and left. Of the 128 individual treatment fractions, 51% and 18% of them show CC baseline drift ≥ 1 and ≥ 3 mm, 30% and 3% show AP baseline drift ≥ 1 and ≥ 3 mm, and 15% and 2% show LR baseline drift ≥ 1 and ≥ 3 mm. Over the entire treatment, 41%, 32%, and 11% of the 37 targets show mean baseline drift < 1 mm in the CC, AP, and LR directions, respectively. In Fig. 6, the variation of tumor motion and its associated baseline drift is shown. Coefficients of Pearson's correlations between the baseline drift and the motion amplitude, and between the baseline drift and the treatment duration, are listed in Table 2. Results presented in bold are statistically significant. Moderate correlation of intrafractional baseline drift and motion amplitude was observed in each motion direction. The reported significance of correlation coefficients in Table 2 is independent of tumor locations.

**Figure 7:**
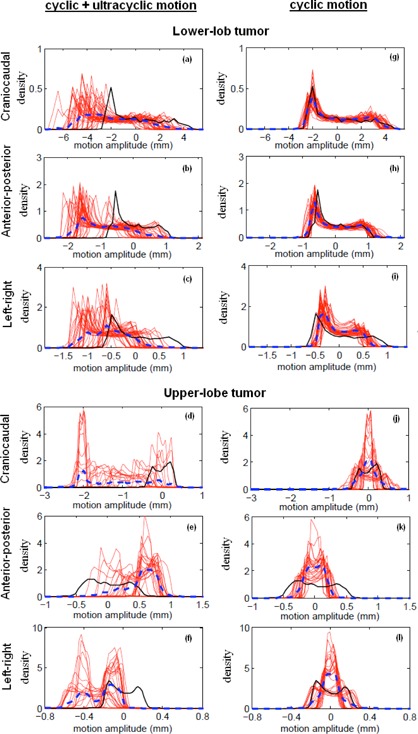
Calculated probability motion functions (pmf) of intrafractional target motion without subtraction of target baseline motion (i.e., ultracyclic plus cyclic target motion (a)‐(f)), and with subtraction of the target baseline motion (i.e., cyclic target motion (g)–(l)), for a lower‐ and upper‐lobe tumor. Each red solid line represents the per‐100 s pmf, and the black solid line is the reference pmf calculated using the first 100 s motion data. The blue dotted line is the averaged pmf of the entire treatment. The upper‐lobe tumor is not only associated with noticeable baseline variation, but also associated with significant variability of target motion amplitude and period. The lower‐lobe tumor, on the other hand, is associated with excellent reproducibility of the pmf in spite of the baseline variations.

**Figure 8:**
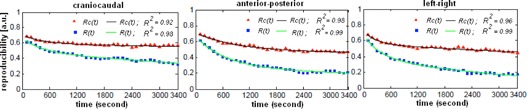
Calculated reproducibility of per‐100 s probability motion functions starting at the 110th second up to the 3400th second (median treatment length of the patient group).The red triangle corresponds to the motion data with subtraction of the ultracyclic baseline (i.e., cyclic motion) and the blue square is the original motion data without subtraction of the baseline (i.e., the cyclic plus ultracyclic motion), respectively. The black and green lines are the two‐exponential fits to the data using the Levenberg‐Marquardt algorithm. $R^{2}$ of the fits are also given.

To illustrate how changing motion amplitude, period, and baseline affects the intrafractional reproducibility of pmf, the per‐100 s pmfs are shown for an upper lobe tumor in Fig. 7. The reproducibility of the per‐100s pmfs is strongly patient‐dependent. As shown in Fig. 7, the pmf of another lower lobe tumor is seen to be highly reproducible once the baseline variation was subtracted from the original motion data. For the entire population, the reproducibility of pmf calculated without and with subtraction of baseline (i.e. R(t) according to Eq. (1) and $R_{c}$(t) according to Eq. (2)) is shown in Fig. 8. It was found that R(t) and $R_{c}(t)$ can be well described by a two‐exponentials function with a fast and slow decay component. Coefficients of the fitted two‐exponentials function, $A_{0}\;\times \;\text{exp}(A_{1}\times \;t)\;+\;A_{2}\;\times \;\text{exp}(A_{3}\;\times \;t\;)$, are given in Table 3. For the R(t), the slow decay component is dominant in each motion direction. For the $R_{c}(t)$, the slow decay component is dominant in the AP motion. For the CC motion, R(t) are 0.57, 0.49, 0.40, and 0.37, and $R_{c}(t)$ are 0.64, 0.61, 0.56, and 0.57 at 5, 10, 30, and 45 minutes, respectively. Differences at the corresponding time intervals are 0.07 (7%), 0.11 (11%), 0.16 (16%), and 0.2 (20%), indicating that the influence of baseline variation increases with time.

**Table 1:** Population statistics of intrafractional baseline variation

	*Craniocaudal (mm)*	*Anterior‐Posterior (mm)*	*Left‐Right (mm)*	*3D (mm)*
GM	‐1.2	‐0.7	0.2	1.8
Σ	1.5	0.7	0.5	1.4
σ	1.9	1.0	0.6	2.3

GM = group mean; Σ = systematic error; σ = random error.

**Table 2:** Correlations of the magnitude of intrafractional baseline drift with the amplitude of intrafractional target motion and treatment time

	*Baseline*		
	*Craniocaudal*	*Anterior—Posterior*	*Lefi—Right*
motion amplitude	**0.42**	**0.37**	**0.36**
time	0.08	**0.26**	0.16

Values are the Pearson's correlation coefficients r, and values presented in bold are statistically significant.

**Table 3:** Coefficients of the two‐exponential fits to the reproducibility of probability motion function (pmf) calculated for the motion data including baseline R(t) and excluding baseline $R_{c}(t)$

	$A_{0}$	$A_{1}$	$A_{2}$	$A_{3}$	$R^{2}$
R(t)					
	1.7E‐01	‐2.4E‐03	4.9E‐01	‐1.0E‐4	0.98
CC	(1.4E‐01, 2.1E‐01)	(‐3.5E‐03, ‐1.2E‐03)	(4.6E‐01, 5.3E‐01)	(‐1.4E‐04,‐8.4E‐05)	
A P	3.9E‐01	‐1.5E‐03	2.8E‐01	‐9.23E‐05	
AP	(3.3E‐01,4.6E‐01)	(‐1.9E‐03,‐1.0E‐03)	(2.0E‐01,3.5E‐01)	(‐1.8E‐05,‐4.5E‐06)	0.99
LR	2.7E‐01	‐2.9E‐03	4.1E‐01	‐2.8E‐04	
LR	(2.3E‐01, 3.2E‐01)	(‐3.9E‐03, ‐1.9E‐03)	(3.7E‐01, 4.5E‐01)	(‐3.2E‐04,‐2.4E‐04)	0.99
$R_{c}(t)$					
	1.2E‐01	‐2.2E‐03	5.9E‐01	‐1.9E‐05	
CC	(8.9E‐2, 1.4E‐01)	(‐3.5E‐03, ‐1.0E‐03)	(5.7E‐01, 6.1E‐03)	(‐3.3E‐05,‐4.2E‐06)	0.92
AP	2.5E‐01	‐9.92E‐04	4.6E‐01	‐1.6E‐06	0.98
AP	(1.5E‐01, 3.4E‐01)	(1.5E‐03, ‐4.7E‐04)	(3.6E‐01, 5.7E‐01)	(‐6.0E‐05,‐5.7E‐05)	0.98
LR	1.7E‐01	‐3.7E‐03	5.6E‐01	‐5.9E‐05	
LR	(1.3E‐01, 2.2E‐01)	(‐5.3E‐03, ‐2.0E‐03)	(5.4E‐01, 5.8E‐01)	(‐7.3E‐05,‐4.6E‐05)	0.96

Values given in brackets are the 95% confidence intervals of the fit coefficients.

CC = craniocaudal motion; AP = anterior‐posterior motion; LR = left‐right motion.

## IV. DISCUSSION

This study analyzed tumor motion data obtained from the log files in CyberKnife real‐time target tracking SBRT. Previous studies that employ 4D simulation/cone‐beam CT provided estimate of tumor location errors and evaluation of motion trajectory at certain time points (e.g., before and after the treatment).^16^, ^17^, ^18^ Continuous fluoroscopic imaging has the potential to track the tumor motion trajectory throughout the treatment, but it raises concerns of additional exposure to the patient, which requires substantial justification.^(^ ^8^ ^,^ ^19^ ^)^ This study inferred the tumor location from the fiducial markers implanted in or near the tumor, and derived the motion of the tumor from continuously updated correlation model based on the external breathing signal and the internal marker position. It is known that correlation between the internal markers and the tumor is not perfect^(20)^ and correlation error between the external breathing signal and the internal markers exists,^(14)^ but the correlation motion data still offers valuable information about the continuous motion of tumor within a time frame relevant to CyberKnife SBRT and beam‐gated SBRT.

For our patient population, the overall cycle‐to‐cycle motion amplitude in the principal motion axis (craniocaudal) is 6.0 ± 4.6 mm (SD), which is close to means of 5.0 ± 1.6 mm and 9.9 ± 6.4 mm reported by Suh et al.^(11)^ and Guckenberger et al.,^(16)^ respectively. Similarly, the overall cycle‐to‐cycle period is 3.6 ± 0.6 s (SD), which is very similar to the mean of three other patient populations (3.7 s).^(^ ^8^ ^,^ ^11^ ^,^ ^20^ ^)^ Unlike the CC motion that shows greater amplitude in lower lobe tumors than in upper and middle lobe tumors, the motion period shows no noticeable dependence of the tumor location. In this work, we found that the exhale length is more variable than the inhale length. This implies that the dose‐weighting scheme of 4D dose accumulation shall have greater uncertainty contributing from the exhale phases.

Previously, Bissonnette et al.^(12)^ found that the inter‐ and intra‐acquisition variability of tumor mobility increased with the averaged motion amplitude. The study also found that the motion amplitude is not only a significant predictor of the intrafractional variability (1 SD) of tumor motion, but also a significant predictor of the magnitude of intrafractional baseline variation in each direction. This suggests that larger PTV margin may be needed for lower lobe tumor in nontarget‐tracking SBRT treatments. Several studies have showed that active breath control and abdominal compression are effective to limit motion amplitude, but these techniques also resulted in greater variability of target position and motion amplitude,^(^ ^12^ ^,^ ^21^ ^)^ and are unnecessary and impractical in target‐tracking SBRT concerning the long treatment time. Therefore, the need of larger margin for lower lobe tumors in conventional SBRT treatments remains, since motion reducing techniques are frequently applied to these tumors with the greatest mobility.

From the results of Fig. 3 and Table 2, neither the variability of cyclic (CC, LR, and 3D) target motion nor the ultracyclic (CC and 3D) baseline drift appears to increase or decrease with treatment time for the scale from 1200 s to 6000 s. As previously demonstrated by Guckenburger et al.^(16)^ using four repeated 4D CT scans at ten‐minute intervals, the intrafractional tumor motion was relatively stable, although significant variations were possible in patients with poor pulmonary function. Bisonnette and colleagues similarly reported that the tumor motion variation was limited to < 1 mm and did not differ by the treatment fraction time. For a series of 129 patients from the United States using 3D CBCT, Shah et al.^(17)^ showed that the intrafractional (3D) baseline drift > 5 mm was significantly more frequent when treatment time was longer (22.6 ± 5.9 min vs. 20.7 ± 4.9 min), and the (3D) baseline drift > 2 mm was found to correlate with longer treatment times. As demonstrated in Fig. 6, the baseline could deviate from its starting position in the middle of the treatment and return to nearly the starting position at the end of the treatment. Therefore, taking samples of the tumor baseline position at the start and at the end of the treatment is likely to exclude significant change in the middle of the treatment, leading to underestimation of the intrafractional baseline drift. For the CC motion, the mean baseline drift estimated using the entire time series and estimated using the first 100 s and the last 100 s (a scenario corresponds to using CBCT) differs by 0.8 mm. Despite the notable differences between this study using motion data from the entire treatment and others using 4D CT or CBCT to derive the baseline position, the calculated statistics of the intrafractional baseline drift is remarkably similar.^(^ ^10^ ^,^ ^16^ ^,^ ^18^ ^)^ The results of Sonke et al.^(10)^ and ours similarly indicated posterior and superior baseline drifts, whereas Guckenberger et al.^(16)^ indicated posterior and inferior drifts. It is unknown whether the directions are associated with the gravity and other physio‐ and psychological processes, such as gradual relaxation of muscle and stress.

Constant motion pattern is the fundamental assumption for most 4D treatment planning strategy. Figure 7 clearly shows that this assumption hardly holds. The reproducibility of pmf is affected by not only the cyclic target motion but also the ultracyclic target baseline drift (Fig. 8). The influence of baseline variation is more important in the first 15 minutes and then gradually diminishes. It is also more important in the AP direction. We found that the reproducibility of population pmf decays following a two‐exponentials function that has a fast and slow decay component. The underlying mechanisms of the fast and slow decay are not fully understood. One possible explanation for the fast decay component is the psychological nervousness of the patient during the beginning of treatment. For the AP motion, the weight of the fast decay component is larger that of the slow decay component in the R(t) curve and becomes smaller in the $R_{c}(t)$ curve. This signifies that the fast decay component is strongly related to the ultracyclic baseline drift. The observed motion reproducibility curve shown in Fig. 8 seems to support our clinical practice of allowing patients to rest on the CT scanner couch for about ten minutes before the 4D CT simulation. Overall, the cyclic target motion is relatively stable over time, with reproducibility over 50% for the AP and LR motions, and 60% for the CC motion up to 50 minutes.

The general approach of 4D planning is to accumulate dose using a patient breathing model. The ultimate question is whether the respiratory phases correspond to the exact spatial location over the fraction as it had been in the simulation 4D CT images. Attempts have been made to incorporate such variability of the pmf into 4D planning, but that requires estimation and generation of error bars associated with the pmf.^(2)^ As demonstrated by Fig. 7, the shape of the pmf can be far more “random” than those used to test the robustness of different probabilistic optimization algorithms.^(^ ^2^ ^,^ ^22^ ^)^ It has been demonstrated that target tracking techniques such as dynamic MLC tracking, robotic respiratory tracking, and tumor trailing strategy based on the moving average algorithm have the ability to adapt the baseline variation, but such adaptation is hardly reflected in the 4D dose calculation. Recently, Chan et al.^(23)^ have demonstrated that the sensitivity of 4D dose calculation to the variation of target motion differed by organs of interest in target‐tracking SBRT, with the greatest dosimetric errors observed in structures that were static or moved asynchronously with respect to the tracking object. Since planning risk volumes (PRV) have the limitations in serially functioning organs such as the spinal cord,^(24)^ we suggest to simulate the intrafractional (and also interfractional) baseline drifts using the population data during treatment planning to avoid potential risks of overdosing these critical organs.

## V. CONCLUSIONS

This study shows that intrafractional target motion, excluding the influence of baseline drift, is relatively stable with reproducibility over 60% in CC and 50% in AP and LR directions within a time frame that is typical of lung SBRT. However, tumor baseline can change substantially in SBRT and effectively reduces the reproducibility of pmf in the majority of patients. These results suggest that methods based on dose convolution technique or explicit 4D dose calculation may only serve as a first‐order approximation to the dosimetric effects of target motion. It may be important to simulate the interfractional in addition to intrafractional baseline drifts to avoid potential risks of normal tissue complications, particularly if tumor is close to the organs at risk.

## ACKNOWLEDGMENTS

This work was partially supported by the Hong Kong Adventist Hospital.
